# Attitudes of nurses, paramedics, and medics towards security prisoners: a cross-sectional study

**DOI:** 10.1186/s40352-024-00275-8

**Published:** 2024-04-27

**Authors:** Liel Hadida, Oren Wacht, Ilana Livshiz Riven, Orli Grinstein-Cohen

**Affiliations:** 1 Department of Nursing, Leon and Matilda Recanati School for Community Health Professions, Faculty of Health Sciences, Ben-Gurion University of the Negev, 1 David Ben Gurion Boulevard, Beer Sheva, Israel; 2 Department of Emergency Medicine Leon and Matilda Recanati School for Community Health Professions, Faculty of Health Sciences, Ben-Gurion University of the Negev, 1 David Ben Gurion Boulevard, Beer Sheva, Israel

**Keywords:** Security prisoners, Theory of Planned Behavior, Cross-sectional study, Nurses, Paramedics

## Abstract

**Background:**

Security prisoners in Israel are those imprisoned due to offenses involving harming state security or from nationalistic motivations. On the one hand, they are accused of a serious criminal offense that harmed state security, while on the other hand they have a right to healthcare like any human being. According to the Theory of Planned Behavior, an attitude is one of three components that predict a behavior intention. The study aims to evaluate the attitudes of nurses, paramedics, and medics toward security prisoners, and to identify factors that could be related to their attitudes.

**Methods:**

A cross-sectional study, conducted using a convenience sample. Attitudes toward security prisoners were measured using the Attitudes Towards Prisoners (ATP) questionnaire. The study included 281 participants. The results show that the nationality of staff members (Jewish, Muslim, or Christian) did not influence their attitudes toward security prisoners (*p* > 0.05).

**Results:**

Staff members who had treated a security prisoner showed a more positive attitude compared with those who had never treated a security prisoner (*p* < 0.05). The study also found that the youngest group of participants (20–30 years) had a lower average attitude compared with older age groups (*p* < 0.05). This may be due to the younger participants’ closer age to the experience of military service.

**Conclusions:**

This study showed that there is no connection between staff members’ nationality and their attitudes toward security prisoners. This indicates that the staff treat patients in accordance with the equality value. By characterizing variables related to the staff’s attitudes we can propose appropriate training programs for the studied staff and the introduction of this topic into the various curricula in Israel, thus improving the quality of staff care.

**Supplementary Information:**

The online version contains supplementary material available at 10.1186/s40352-024-00275-8.

## Introduction

Security prisoners in Israel are those imprisoned due to offenses involving harming state security or from nationalistic motivations. Security prisoners are an ambivalent subject in today’s Israeli society: On the one hand, they are accused of a serious criminal offense that harmed state security (such as planning or performing a terrorist attack, a stabbing, kidnapping soldiers, and so on), while on the other hand they have a right to health like any human being, and the State of Israel must care for their health and provide them with appropriate medical care when they require it (Prison Service, 2019).

It is important to study the attitudes of nurses, paramedics, and medics regarding security prisoners, because according to the Theory of Planned Behavior, an attitude is one of three components that motivate behavior (Ajzen, 1991). When we can characterize their attitudes, we will be able to estimate their intention (or avoidance) regarding treating security prisoners.

### Aim

The purpose of this study is to examine the attitudes of nurses, paramedics, and medics and to identify variables that could influence their position.

This involved evaluating the attitudes of nurses, paramedics, and medics toward security prisoners and to identify demographic variables that could influence their attitudes. The research literature contains insufficient information regarding the attitudes of healthcare staff toward prisoners in general, and in particular regarding the attitudes of nurses, paramedics, and medics toward security prisoners. The conclusions of this study may contribute to understanding whether there is a need for training that will improve the quality of treatment the staff provides, regardless of the patient’s legal background, as stated in the Patients’ Rights Law (Ministry of Health, 1996).

## Background

### Security prisoners

A prisoner is a person imprisoned for an offense and legally held in the prison’s custody (Ministry of Internal Security, 1971). The population of prisoners in Israel is over 17,000 (Prison Service, 2019). Among these, as of March 2024, there are 8,900 security prisoners (Prison Service, 2024). In Israel, there is a division into criminal prisoners and security prisoners. A security prisoner is defined as “A prisoner who was convicted and sentenced due to committing an offense, or arrested under suspicion of an offense, which by its nature or circumstances is defined as a clear security offense, or when the motivation for committing it was nationalistic”. The population of security prisoners in Israel is between 4,500 and 5,500 prisoners, almost one third of the population of prisoners in Israel (Prison Service, 2019; Addameer, 2021).

Prisoners’ rights are established in the Patients’ Rights Law. The legal past of those who have committed an offense is not supposed to influence the quality of treatment they receive: “A caregiver or a medical institution shall not discriminate between patients for reasons of religion, race, sex, nationality, country of origin, sexual inclination, age, or any other such reason” (Ministry of Health, 1996, 2–3).

### Healthcare for prisoners

The main healthcare providers in Israeli prisons are doctors and medics (State Comptroller, 2015, 6–7). Paramedics and medics are at the front line required to provide urgent care in the field, such as the sites of terrorist attacks, where they are required to treat future prisoners. Many cases of prisoners with medical problems are taken from the prisons to hospitals in ambulances staffed by paramedics and medics (Mason et al., 2013). The nursing roles are given little attention in the overall medical care of security prisoners. This despite the fact that nursing staff often encounters prisoners in medical centers, such as emergency departments, hospital wards, in the community, and in the legal department of mental health hospitals. It is apparent that the role of legal nurses in Israel requires definition and promotion, and that nurses, paramedics, and medics should be given training to handle patients with a legal background in order to provide them with quality, beneficial care.

Prisoners in Israel are entitled to medical care according to a physician’s instructions. Prisoners are also entitled to the basic medical services given by healthcare funds. “The services included in the basic basket: medical check-ups, treatment, deciding upon medical care and definition of living conditions, employment, and food menu for sick prisoners. Medical follow-up and supervision for prisoners diagnosed as chronically sick. Information and guidance on education for hygiene and health, and more” (IDF Chief Medical Officer, 2019).

According to the ethical standards accepted in the western world and in the American Nursing Association, nurses are required to respect the individuality of every patient (Association, 2001). In Israel, too, one of the most important principles of the Ethical Code for Nurses in Israel is the principle of equality and fairness, meaning “the right to receive care without discrimination” (Israeli Nurses Association, 2018, 3). At the same time, it appears that this principle is difficult to observe. Nurses treating prisoners report their awareness that they need to treat the patients professionally and objectively, but at the same time they were aware that knowing the patients’ criminal past disrupted the quality of care they gave them (Dhaliwal & Hirst, 2016; White & Larsson, 2012).

### Neutrality and Humanitarian Healthcare

The principle of neutrality in medicine guarantees neutral, humanitarian medical care for patients, unrelated to politics. This principle has been adopted by healthcare professionals and organizations. According to this ideology, healthcare services and staff are expected to uphold the principle of objectivity and non-discrimination on the basis of nationality, race, gender, religious belief, status, or political opinion (OCHA, 2010). The principle of medical neutrality is embedded in international humanitarian laws, such as the Geneva Convention and the Hague Convention, guaranteeing unbiased humanitarian aid even in the contexts of violence and war between different groups (Esgain & Solf, 1962; Moorehead, 1998). While the Geneva Convention provides guidelines for medical care of injured and sick enemy fighters and prisoners of war, there are no similar instructions for the treatment of terrorists. Medical care of terrorists creates extreme situations where acts of violence and the protection of human rights clash, which can lead to serious ethical dilemmas (Merin et al., 2015).

The duty to provide care for patients who have committed violent nationalistic crimes raises ethical dilemmas in the caregivers: On the one hand, according to the principles of equality and objectivity, they should be treated like any other person, and they deserve good medical care (Gesundheit et al., 2009); On the other hand, some studies emphasize the various dilemmas related to providing medical care to terrorists. Caregivers around the world find themselves treating injured victims alongside the perpetrators of terrorist attacks, and this reality creates ambivalence among medical staff (Davis, 2009; Gross, 2013; Merin et al., 2015). All patients reaching hospital are supposed to be unambiguously equal, without exception. However difficult it may be, the medical staff must not be judgmental. Punishment is not the role of the medical staff; their duty is to preserve the patients’ lives and care for them. Judgment should be the exclusive domain of the legal system, while medical staff should engage in treatment without bias and with a clean conscience (Merin et al., 2015).

### Attitudes

The current study examined the attitudes of nurses, paramedics, and medics toward security prisoners. An attitude is a general and relatively permanent evaluation a person has toward subjects or people. Attitudes can range from positive to negative, and are important because they influence the way people and objects are treated and the way people behave in social situations. Attitudes are built through learning and cognitive processes of creating organized internal order in our thinking (Rocks & Schwerlwaltzeld, 2000). A common definition of an attitude is a mental tendency to act resulting from an evaluation of a specific subject as preferred or rejected (Ajzen, 2001).

The research literature contains many theories claiming that attitudes lead to behavior (Doob, 1947; Fazio, 1986). The leading one is Ajzen’s Theory of Planned Behavior (Ajzen, 1985, 1991), which postulates that a particular behavior depends on the degree of intention to perform this behavior, and can be explained by three interrelated variables: (1) Attitudes toward the behavior (positive or negative); (2) Subjective norms representing society’s expectations regarding the behavior; (3) The sense of ability to perform the behavior. Thus, if individuals have a positive attitude toward a particular behavior, they believe that social norms encourage it, and they perceive their self-efficacy toward it as positive, they will have an intention to perform this behavior, which in turn will lead to actually performing the behavior (Ajzen, 1985, 1991). The current study deals with the attitude level, one of the three motivations for behavior, according to Ajzen’s approach. The study examined the attitudes of nurses, paramedics, and medics toward security prisoners, and therefore, the hypothesis, based on Ajzen’s theory, is that these attitudes would indicate behaviors of caring for prisoners or avoiding caring for them.

### Nurses’ attitudes to prisoners

Nurses play an important role in treating criminals due to the various challenges this treatment creates. Many nurses feel uncomfortable treating a person who has injured, raped, or murdered other people. They find it difficult to be empathic and feel great insecurity during the treatment (Astari & Yuliatun, 2020). Many studies focus on the role of nurses working in prisons and the experience of treating prisoners (Flanagan & Flanagan, 2001; la Cerra et al., 2017; Powell et al., 2010; Weiskopf, 2005), but very few studies have examined the attitude of the caregiving nurses toward prisoners. One of them is a qualitative study conducted in Atlanta in 2019, analyzing the attitudes of nurses in prisons regarding the challenges of treatment and the use of the person-focused treatment model. In this study, the nurses reported that knowing the prisoner’s criminal record influenced their care of the prisoner, and argued that compassionate treatment without judgement is required. They also noted the importance of developing objective treatment. They argued that holistic treatment, focusing on the person, is justified when caring for prisoners, both legally and ethically (Solell & Smith, 2019).

An older study from 1997 examined the attitudes of nurses in prisons toward prisoners and found that age is a variable that influences the attitudes of nurses: older nurses showed a more positive attitude toward prisoners compared with younger nurses. The study also raised the possibility that education is another variable that could influence positive attitudes, but that more research was required in order to examine whether education above a bachelor’s degree is connected to a more positive attitude toward prisoners (Shields et al., 1997).

### Security prisoners

Our study examines the attitudes of staff toward security prisoners, who are usually associated with perpetrating acts of terrorism. During times of terrorist attacks, citizens’ basic sense of security is threatened, there is a fear of total annihilation, and memories of previous terror attacks are awakened (Tummala-Narra, 2005). Healthcare professionals experience similar reactions to those of the civilian population, and they also have to handle as part of their professional role painful and almost unbearable situations that trigger extreme emotional responses (Krajewski, 2002; Ofran & Giryes, 2004). A study examining the response of healthcare workers during periods of suicide and shooting attacks found that nurses worked under great stress and fear (Riba & Reches, 2002). Another study found that a discriminatory response toward some of the patients is not an impossible scenario, and that the equal approach to all patients is not taken for granted in light of the clash between the duty of care and the emotions regarding patients who have perpetrated acts of terrorism. The study showed that some of the nurses considered the patients’ actions as reasons to refuse them nursing care or to postpone such care. It found that certain patients can awaken a clash with the nurses’ beliefs, so that nurses do not provide identical nursing care to such patients (Margalith et al., 2008).

## Method

### Design

A cross-sectional study. The sample included 281 participants, of whom: 130 nurses, 76 paramedics, and 75 medics. The sample was conducted on behalf of the Health Science Faculty, Ben-Gurion University. After approval was obtained for conducting the study, the questionnaire was distributed in March 2021 over social media such as Facebook and WhatsApp groups belonging to nurses, paramedics, and medics, using the snowball method. The questionnaire was available for two weeks. The Qualtrics software platform was used for the online questionnaire. The questionnaire had quantitative and qualitative parts, and in this article we will refer only to the quantitative part.

### Participants and Research Context

There were 402 participants in the study. A non-inclusion criterion was set whereby participants who answered under 80% of the questionnaire would be excluded. After filtering, 281 participants remained, who constituted the sample. Table 1 below describes the sample population characteristics.

**Table 1:** Description of the sample characteristics (*N* = 281)

Characteristic	Values	N	%
Sex	Female Male	158 123	56.2 43.8
Nationality	Jewish Arab (Muslim or Christian)	248 33	88.3 11.7
Age group distribution	20–30 31–40 41–50 51–60	105 57 73 45	37.4 20.3 26 16
Profession	Paramedic Medic Nurse	76 75 130	27 26.7 46.3
Professional experience (years)	0–5 6–10 11–20 20<	113 51 49 67	40.2 18.1 17.4 23.8
Paramedic education	Ambulance course Military paramedic course Hospital course Bachelor’s degree in emergency medicine Other bachelor degree Master’s degree Ph.D.	26 24 9 7 15 2 1 0	6.6 6.1 2.3 1.8 3.8 0.5 0.3 0
Medic education	No education Bachelor’s degree Master’s degree In paramedic course Emergency medicine student	1 31 7 3 1	0.3 7.9 1.8 0.8 0.3
Nurse education	Practical Registered Bachelor’s degree Master’s degree Ph.D.	17 44 44 1 50	4.3 11.3 11.3 0.3 12.8
Have you treated a security prisoner at work?	Yes No	172 109	61.2 38.8

*Note: Where results do not add up to 100%, this is because one or more participant did not answer this question*

Table 1 shows that most of the participants were women (56.2%), Jewish (88.3%), aged 20–30 (37.4%), and with work experience of 0–5 years (40.2%). Most of the participants reported having treated a security prisoner at work (61.2%). Of them, 67.2% reported treating security prisoners 0–5 times, 10.6% reported treating security prisoners 6–10 times, and 22.2% reported treating security prisoners over 10 times.

#### Attitudes to prisoners

The original questionnaire, Attitudes Towards Prisoners (ATP), was developed in 1985 by Prof. Melvin and colleagues (Melvin et al., 1985). This is a 36-item questionnaire examining attitudes toward prisoners. Each question is ranked on a 5-point Likert scale from “completely disagree” to “completely agree”. Some previous studies have focused on particular sub-categories of prisoners, such as: sex offenders, young prisoners, etc. (Chui & Cheng, 2019). This study focused on the sub-category of security prisoners.

The research team translated the questionnaire from English to Hebrew using the “back and forth” method. The questionnaire was first given as a pilot to 5 staff members to test that it appeared correctly on the software. After corrections, it was given to the participants. The pilot results were not included in the study’s results.

The research tool showed good measures of reliability in the studies that used it: test-retest (*r* = 0.82) and split-half (*r* = 0.84–0.92) in the study by Melvin and colleagues (Melvin et al., 1985). In addition, the Cronbach alpha was in the range between 0.88 (Kjelsberg et al., 2007) and 0.95 (Ortet-Fabregat et al., 1993). The questionnaire had already been validated. The tool has been used with many groups of participants and was found to have construct validity. In the current study, the Cronbach alpha was α = 0.842.

#### Demographic questionnaire

The participants filled in a 10-item questionnaire with questions about demographic variables such as: sex, age, nationality, professional, years of experience, education, and experience in caring for security prisoners.

### Statistical analysis

We examined the connection between the variable “attitude toward security prisoners” and the variables: education level, years of experience, and religion using the One-Way ANOVA test. We examined the connection between the variable “attitude toward security prisoners” and the variable: experience in caring for a security prisoner using the t-test for independent samples. We built a multivariate linear regression model for predicting the attitude toward security prisoners. The model contained the dependent variable “attitude toward security prisoners” and the variables found to be significantly connected to behavior in the univariate analysis, and also variables that had theoretical background to assume that they might predict positive attitudes. The statistical data analysis was conducted using IBM SPSS Statistics version 26. For all tests, the p-value under 0.05 was considered statistically significant.

## Results

In order to construct the variable “attitudes toward security prisoners”, we calculated each participant’s average answer to 36 items on the attitudes questionnaire. The items were ranked on a 1–5 scale, with 1 – “completely disagree” and 5 – “completely agree”. Three items (“security prisoners never change”, “it is not wise to trust a security prisoner too far”, and “most security prisoners are too lazy to earn an honest living”) were removed from the statistical analysis because they significantly detracted from the tool’s reliability. After removing these items, the Cronbach alpha was α = 0.842. All the data are presented after performing the score reversal where required.

**Table 2:** Distribution of items – Attitudes toward security prisoners

	1 (%)	2 (%)	3 (%)	4 (%)	5 (%)	Mean ± Standard deviation
* Security prisoners are different from most people	4.9	16.9	14.1	22.3	13.3	3.31 ± 1.21
Only a few security prisoners are really dangerous	9.2	24.6	25.6	13.6	2.8	2.68 ± 1.02
* Security prisoners never change	2.8	13.6	19.9	24.6	9.2	3.33 ± 1.05
Most security prisoners are victims of circumstance and deserve to be helped	6.9	21	24.8	13.8	4.6	2.83 ± 1.05
Security prisoners have feelings like the rest of us	14.1	20.5	17.6	15.6	1.8	2.57 ± 1.12
* It is not wise to trust a security prisoner too far	20.2	35.8	7.4	4.1	3.3	2.07 ± 1.02
I think I would like a lot of security prisoners	2	10.2	13.8	29.4	14.1	3.62 ± 1.05
Bad prison conditions just make a security prisoner more bitter	27.6	25.3	14.6	3.6	0.3	1.92 ± 0.9
* Give a security prisoner an inch and he’ll take a mile	2.8	17.9	19.2	16.6	14.3	3.3 ± 1.16
* Most security prisoners are stupid	11.3	19.7	20.2	15.6	4.6	2.75 ± 1.15
Security prisoners need affection and praise just like anybody else	28.6	35	5.1	1.5	0.5	1.73 ± 0.74
* You should not expect too much from a security prisoner	6.1	29.2	12.8	13.6	9.7	2.88 ± 1.21
* Trying to rehabilitate security prisoners is a waste of time and money	10.2	28.1	16.9	13.6	2	2.56 ± 1.04
* You never know when a security prisoner is telling the truth	9.7	14.1	15.6	20.2	11	3.12 ± 1.28
Security prisoners are no better or worse than other people	1.5	9.7	19.4	29.7	11	3.54 ± 0.98
* You have to be constantly on your guard with security prisoners	3.8	17.6	12	25.8	11.3	3.32 ± 1.17
In general, security prisoners think and act alike	1.3	9	11.5	33.8	15.9	3.75 ± 0.99
If you give a security prisoner respect, he’ll give you the same	6.6	25.8	19.4	15.9	3.3	2.76 ± 1.04
* Security prisoners only think about themselves	7.7	33	18.9	8.4	3.1	2.52 ± 0.98
There are some security prisoners I would trust with my life	5.9	34.8	18.4	7.9	3.6	2.55 ± 0.97
Security prisoners will listen to reason	28.9	23.5	14.6	3.6	0.3	1.9 ± 0.91
* Most security prisoners are too lazy to earn an honest living	3.8	28.6	22.3	12.3	3.3	2.75 ± 0.96
* Security prisoners are just plain mean at heart	2.3	4.6	17.4	33	12.8	3.7 ± 0.95
* Security prisoners are always trying to get something out of somebody	5.9	9.5	13.6	31.2	10.5	3.43 ± 1.14
The values of most security prisoners are about the same as the rest of us	5.6	25.8	23	12.3	3.1	2.73 ± 0.98
Most security prisoners have the capacity for love	15.1	25.8	14.1	12	3.3	2.46 ± 1.14
* Security prisoners are just plain immoral	8.4	39.6	15.1	5.4	1.8	2.32 ± 0.87
* Security prisoners should be under strict, harsh discipline	6.9	18.2	17.1	23.8	4.1	3 ± 1.1
* In general, security prisoners are basically bad people	16.1	29.9	13.8	7.9	2.3	2.29 ± 1.04
Most security prisoners can be rehabilitated	8.4	32.5	17.6	6.9	4.9	2.53 ± 1.05
Some security prisoners are pretty nice people	8.7	19.9	24	14.6	3.1	2.76 ± 1.05
I would like associating with some security prisoners	4.1	8.7	21.5	29.4	6.4	3.36 ± 1
* Security prisoners respect only brute force	0.3	4.1	8.2	22.3	35.5	4.26 ± 0.9
If a security prisoner does well in prison, he should be let out on parole	7.2	34.3	17.1	6.6	4.9	2.54 ± 1.03
* I would prefer to avoid treating a security prisoner if I had the choice	0.5	3.8	15.1	25.3	24.8	4 ± 0.92
I feel comfortable treating security prisoners just like any other population	12.3	21.5	10	15.6	10.7	2.87 ± 1.35

* Reversed score items. The data are presented after score reversal.

Table 2 shows that the item with which the participants expressed the highest agreement was “In general, security prisoners think and act alike” (mean 3.75 ± 0.99). The items with which the participants expressed the lowest agreement was “Security prisoners respect only brute force” (4.26 ± 0.9 after reversal).

**Table 3:** Indices of center and distribution of the variable attitudes toward security prisoners (general and split by sectors)

	Average	Standard deviation	Range	Frequent	Median	Total
Total Sample	**2.91**	**0.28**	**1–5**	**3**	**2.9**	**281**
Paramedic	2.9	0.16	2.55–3.27	3	2.9	76
Medic	2.92	0.12	2.61–3.21	2.94	2.93	75
Nurse	2.89	0.11	2.58–3.33	2.88	2.9	130

Table 3 shows that the average attitudes variable is 2.91 (SD = 0.28), the frequent value is 3, and the median is 2.9. It shows that medics have the highest attitude score (positive) toward security prisoners among the studied groups (2.92, SD = 0.28), followed by paramedics (2.9, SD = 0.16), and finally nurses (2.81, SD = 0.11).

### Connections between the variable attitude toward security prisoners and staff’s education, seniority, nationality, age, sex, and treatment experience

No connection was found between the variable attitude toward security prisoners and the variables: education, seniority, nationality, age, sex (*p* > 0.05). Significant differences were found in attitudes toward security prisoners between staff with and without treatment experience (t_278_ = 1/93, *p* < 0.05). Staff who reported that they had provided care for a security prisoner expressed more positive attitudes toward them (average 2.92 compared with 2.88, respectively).

We also studied the differences between different age groups and attitudes toward security prisoners.

**Table 4:** Results of T-tests to examine the differences between age groups in attitude toward security prisoners

Variable	Group	N	Average	SD	F	p
**Attitudes**	20–30 31–40 41–50 51–60	105 57 73 45	2.88 2.91 2.94 2.9	0.13 0.12 0.12 0.14	3.04	0.029*

Table 4 shows that there are significant differences between the age groups regarding attitudes toward security prisoners (F_(278)_ = 3.04, *p* < 0.05). The most positive attitudes were observed in the age group 41–50 (2.94 ± 0.12). Follow-up Scheffe tests found a significant difference in attitudes only between the age group 20–30 and the age group 41–50 (*p* < 0.05). Participants in the age group 41–50 expressed more positive attitudes toward security prisoners than the age group 20–30 (average 2.94 compared with 2.88, respectively).

## Discussion and conclusions

### Treatment experience with security prisoners was related to more positive attitudes

Significant differences were found in attitudes toward security prisoners between staff members with and without treatment experience (*p* < 0.05). Staff members who reported that they had cared for a security prisoner during their work expressed more positive attitudes toward them compared with those who had not cared for such prisoners (average 2.92 compared with 2.88, respectively). Studies have indicated that direct experience with a certain object may create a stronger, more defined attitude than lack of experience with this object (Fazio & Zanna, 1978). The literature also agrees that past experience influences the formation of attitudes and behavior. Fishbein (1979) suggested that behavioral experience provides feedback that might change attitudes but does not directly influence future behavior. The Triandis model suggests that past experience is a direct and decisive cause of behavior, and it also mediates in the formation of attitudes (Landis et al., 1978).

### Is military service related to the staff’s attitude?

A significant difference was found between the attitudes of the 20–30 age group and the 41–50 age group only (*p* < 0.05). The participants in the 41–50 age group expressed more positive attitudes toward security prisoners compared with the 20–30 age group (average 2.94 compared with 2.88, respectively). This finding is supported by the literature, and a study by Shields and colleagues found that older age is related to a more positive attitude toward prisoners compared with younger age (Shields et al., 1997). An explanation can be suggested for the connection between younger age and more negative attitudes: military service in the Israeli Defense Forces, which is compulsory for all Israeli citizens from the age of 18. In other words, the closer the age of the staff to the experience of military service, the more negative their attitudes toward security prisoners, who are usually considered the enemies of the State of Israel.

### Staff’s nationality and education were not found to be related to their attitudes

Another hypothesis tested was the connection between the staff’s religion and nationality and their attitudes towards security prisoners. The sample contained a similar percentage of Jewish and Arab (Muslim and Christian) participants to their proportion in the general Israeli population. No connection was found between the staff’s nationality, whether Muslim, Jewish, or Christian, and their attitudes (*p* > 0.05). This result is encouraging, because it indicates that the staff intends to provide care in accordance with the Patients’ Rights Law: without discrimination and without reference to the patient’s characteristics (Ministry of Health, 1996, 2–3). Staff put aside their personal characteristics, opinions, and emotions. A possible explanation for this result is “emotional detachment” by the caregiving staff, meaning that the staff employ defense mechanisms such as suppression and repression of emotions, and put aside their opinions and emotions (Lazarus, 1993). Medical staff are required to handle distressing situations daily, along with the burden of hard, exhausting work. Emotional detachment can serve the staff as a coping strategy when providing care for terrorists and security prisoners. It enables the caregivers to reduce their emotional involvement with the patients and to establish medical care on professionalism and detachment. Emotional detachment is perceived negatively, as an indication of losing the human contact in caregiving, considering that healthcare professionals are expected to provide patients with attention, compassion, and empathy (Hojat, 2018). Communication skills are considered an important component of medical care, and empathy in particular is perceived as an essential component of the relationship between medical staff and patients. In fact, emotional detachment can be viewed as an aspect of social detachment, as a result of employing passive coping strategies to handle stressful events (Lazarus, 1993). A study by Keshet and Popper-Giveon (2020) found that medical staff treating terrorists may detach from the event and minimize its importance in order to cope with the situation.

In the qualitative study, a Jewish participant said the following: “I treated a terrorist who had perpetrated a stabbing attack on a bus in Tel-Aviv and was shot while being pursued. The terrorist cried and begged for mercy for his life. I gave him complete care as expected from medical staff, but inside it was very difficult. In particular, I found it difficult to express empathy toward him, when the other ambulances were treating citizens who had been stabbed and whom he had intended to injure and kill. This was a very difficult, heart-rending situation…”.

### “Security prisoners think and act alike”

An analysis of the questionnaire’s findings shows that the statement with the highest level of agreement is “In general, security prisoners think and act alike” (average 3.75 ± 0.99). This statement demonstrates stereotypical thinking by the staff, who agree that security prisoners think and act in a similar way, meaning that security prisoners are a homogeneous group with similar characteristics. Stereotypes develop as part of social and cognitive behavior, when the environment shapes beliefs regarding others, particularly regarding the way people identify others and classify them into groups (Lineweaver et al., 2017). A possible explanation for stereotypes is that they save cognitive resources by accessing existing group patterns that are already stored in long-term memory, thus simplifying the perception of people (Macrae et al., 1994). While stereotypes can simplify perception, such categorical thinking is problematic for two reasons: first, stereotypes enable little consideration of the individuality or heterogeneity of group members; and second, negative stereotypes or attitudes can lead to negative behavior toward others (Fazion, 1986; Glasman & Albarracin, 2006; Kraus, 1995).

### Do security prisoners need affection and praise?

In contrast, one of the statements that received relatively low agreement was “Security prisoners need affection and praise just like anyone else” (average 1.73 ± 0.74). 64% of respondents to this question chose “completely disagree” and “disagree”. This statement presents the staff’s difficulty in showing empathy expressed in affection and praise for their patients who are security prisoners. Human beings are social creatures who need intimate relationships in order to survive and thrive. An inseparable part of people’s physical and emotional wellbeing is the need for affection, a positive emotion of belonging and closeness with another person or object, or a feeling of identification and closeness with another person, sometimes interpreted as compassion (Floyd et al., 2021). This result matches the findings in the literature, that nurses have great difficulty in separating between the crime committed by prisoners and the medical care they provide. This difficulty is expressed in nurses’ inability to be empathic and caring toward prisoners, even though they know that this harms their nursing practice and care for the prisoners (Dhaliwal & Hirst, 2016). Quotations from the qualitative study demonstrated the nurses’ empathy toward the security prisoner patients: “He received care just like any other patient, and because he was suffering severe pain due to kidney stones, I felt quite a bit of empathy toward him”. “It wasn’t easy, but I preferred not to know what he had done, but to focus on giving him the best treatment, and I thought about the fact that we have all been created in God’s image…”.

### Statements identified as ambivalent

The statements identified with great ambivalence from the participants (they chose the answer “difficult to decide”) were: “Most security prisoners are victims of circumstance and deserve to be helped” and “Only a few security prisoners are really dangerous” (25% and 26%, respectively, among respondents to these statements). This can indicate the participants’ inability to express a decisive position: Are security prisoners victims and the crimes they committed actually the result of their education, where they grew up, and their life circumstances? Or are security prisoners capable of making decisions and this is the way of life and ideology that they chose? In addition, it is difficult to decide whether security prisoners are “really dangerous” as a result of the crime they committed.

### Researchers’ recommendations

This study could serve as a corner stone for further research in Israel regarding attitudes toward security prisoners. The researchers recommend follow-up studies examining attitudes and also knowledge about security prisoners and behavior related to their treatment. We recommend using a larger appropriate sample size, and perhaps adding more caregiving sectors, such as physicians. It is worth examining the potential influence of experience of terror events and military service on attitudes, and directly comparing staff who have and have not treated security prisoners.

The contribution of this study can be applied by characterizing the variables related to the staff, such as age and treatment experience, educators in healthcare professions can offer workshops to healthcare staff and students. These would deal with issues of equality and discrimination in emergency situations and everyday life. This issue can be raised in department meetings, emphasizing that the ethical code should serve as a guideline for decision making and critical thinking. In addition, the issue of care for security prisoners can be included as an essential component of training nurses, paramedics, and medics through simulations allowing the students to express their fears and emotions.

### Possible changes in attitudes following the 7 October 2023 attack

This study was conducted prior to the war that started with the Hamas terrorist attack on Israel on 7 October 2023. Arrested terrorist suspects who had participated in the attack were brought to Israeli hospitals for medical treatment. As stated above, they are entitled to receive medical care like any other person, and the State of Israel is required to care for their health and give them appropriate medical treatment as required (Prison Service, 2019). Those who did not require hospitalization were treated within the Prison Service facilities. When those who required hospitalization were brought to Israeli hospitals and treated alongside Israeli patients, a public outcry arose and some staff called for these security prisoners not to be treated (Yanko, 2023). On the other hand, there were other opinions, like that of a senior physician in a major Israeli hospital, who said in an interview: “Our duty to provide medical care is embedded in our moral, professional, and humane DNA as medical staff. When terrorists come in, the decision should be unambiguous: treat. The court can decide what to do with them later” (Manhardt, 2023). We believe that had this study been conducted soon after the 7 October 2023 attack, the results would have been different and perhaps the attitudes toward treating security prisoners would have been more negative.

### Limitations

#### Methodological limitations

Cross-sectional study using a convenience sample: The main limitation of a cross-sectional study is that is cannot prove causality and only indicates a connection. This is the first study in Israel to examine attitudes toward security prisoners using questionnaires, and so we chose to use a convenience sample that does not necessarily represent the entire population.

Recruitment of participants through social networks: We have no control over who fills in the questionnaire and there is no way of verifying the truth of the answers. However, the sample seems to be a good representation of the population.

While an examination of staff attitudes cannot always be generalized to predict their behavior in practice, the study still provides a good picture of their intentions.

#### Additional limitations

As noted, the study examined attitudes toward “security prisoners”, who are usually identified as Muslims who committed crimes against the state of Israel, but this is not necessarily true. There is a minority of security prisoners who are Jews who committed crimes against Arabs (unofficial sources estimate that there are around 20 Jewish terrorists in prison). In order to handle this limitation, we noted in the letter requesting participation in the study a clear definition of security prisoners, without mentioning nationality: “A security prisoner is one imprisoned for an offense involving harming national security or from nationalistic motivations”.

### Electronic supplementary material

Below is the link to the electronic supplementary material.

Supplementary Material 1

Supplementary Material 2

Supplementary Material 3

Supplementary Material 4

## Data Availability

All data generated or analysed during this study are included in this published article.

## References

[CR1] Addameer (2021). *Addameer Statistics*. https://www.addameer.org/statistics/ Accessed 7 November 2021.

[CR2] Ajzen, I. (1985). From intentions to actions: A theory of planned behavior. In J. Kuhl, & J. Beckman (Eds.), *Action Control: From cognition to Behavior* (pp. 11–39). Springer.

[CR3] Ajzen I (1991). The theory of planned behavior. Organizational Behavior and Human Decision Processes.

[CR4] Ajzen I (2001). Nature and operation of attitudes. Annual Review of Psychology.

[CR5] Association, A. N. (2001). *Code of Ethics for nurses with interpretive statements*. Nursesbooks. org.

[CR6] Astari AM, Yuliatun L (2020). Nurses’ experience of caring for criminals at emergency department. The Malaysian Journal of Nursing (MJN).

[CR7] Chui WH, Cheng KK (2019). Validation of a Chinese version of the attitudes toward prisoners Scale. The Prison Journal.

[CR8] Davis M (2009). Terrorists are just patients. The American Journal of Bioethics.

[CR9] Dhaliwal K, Hirst S (2016). Caring in correctional nursing: A systematic search and narrative synthesis. Journal of Forensic Nursing.

[CR10] Doob LW (1947). The behavior of attitudes. Psychological Review.

[CR11] Esgain, A. J., & Solf, W. A. (1962). Geneva Convention Relative to the Treatment of Prisoners of War 1949: Its Principles, Innovations, and Deficiencies, *The NCL Rev*, 41.

[CR12] Fazio RH (1986). How do attitudes guide behavior. Handbook of Motivation and Cognition: Foundations of Social Behavior.

[CR13] Fazio RH, Zanna MP (1978). Attitudinal qualities relating to the strength of the attitude-behavior relationship. Journal of Experimental Social Psychology.

[CR14] Fishbein, M. (1979). *A Theory of Reasoned Action: Some Applications and Implications* Nebraska Symposium on Motivation. 7242751

[CR15] Flanagan NA, Flanagan TJ (2001). Correctional nurses’ perceptions of their role, training requirements, and prisoner health care needs. Journal of Correctional Health Care.

[CR16] Floyd K, Morman MT, Maré J, Holmes E (2021). How americans communicate affection: Findings from a representative national sample. Communication Quarterly.

[CR17] Gesundheit B, Ash N, Blazer S, Rivkind AI (2009). Medical care for terrorists—to treat or not to treat?. The American Journal of Bioethics.

[CR18] Glasman LR, Albarracin D (2006). Forming attitudes that predict future behavior: A meta-analysis of the attitude-behavior relation. Psychological Bulletin.

[CR19] Gross ML (2013). Military medical ethics: A review of the literature and a call to arms. Cambridge Quarterly of Healthcare Ethics.

[CR21] Hojat, M. (2018). *Empathy in Health Professions Education and Patient Care*. Springer, 2016.

[CR22] IDF Chief Medical Officer (2019). *Medical Care of Prioners*. *Prison Service Order no*. 04.44.00 [Hebrew].

[CR23] Israel Nurses Association *Ethical Code for Nurses in Israel*., 3. http://hy.health.gov.il/_Uploads/dbsAttachedFiles/300418.pdf [Hebrew].

[CR24] Keshet Y, Popper-Giveon A (2020). Experiences of jewish and arab healthcare practitioners treating terrorists in Israel. Violence and Victims.

[CR25] Kjelsberg E, Skoglund TH, Rustad A-B (2007). Attitudes towards prisoners, as reported by prison inmates, prison employees and college students. Bmc Public Health.

[CR26] Krajewski B (2002). Emergency nurse’s response to terrorism. International Journal of Trauma Nursing.

[CR27] Kraus SJ (1995). Attitudes and the prediction of behavior: A meta-analysis of the empirical literature. Personality and Social Psychology Bulletin.

[CR28] la Cerra C, Sorrentino M, Franconi I, Notarnicola I, Petrucci C, Lancia L (2017). Primary care program in prison: A review of the literature. Journal of Correctional Health Care.

[CR29] Landis D, Triandis HC, Adamopoulos J (1978). Habit and behavioral intentions as predictors of social behavior. The Journal of Social Psychology.

[CR30] Lazarus, R. S. (1993). Coping theory and research: Past, present, and future. *Fifty Years of the Research and Theory of RS Lazarus: An Analysis of Historical and Perennial Issues*, 366–388.

[CR31] Lineweaver TT, Roy A, Horth M (2017). Children’s stereotypes of older adults: Evaluating contributions of cognitive development and social learning. Educational Gerontology.

[CR32] Macrae CN, Stangor C, Milne AB (1994). Activating social stereotypes: A functional analysis. Journal of Experimental Social Psychology.

[CR33] Manhardt, Y. (2023). A doctor who treated a terrorist: It’s the essence of evil, but it’s a duty to treat everyone. *Ynet* (15 Oct. 2023). https://www.ynet.co.il/health/article/b1pii4tbt [Hebrew].

[CR34] Margalith I, Tabak N, Granot T (2008). Student nurses’ care of terrorists and their victims. Nursing Ethics.

[CR35] Mason C, Burke TW, Owen SS (2013). The dangers of transporting ailing inmates. Corrections Today.

[CR36] Melvin KB, Gramling LK, Gardner WM (1985). A scale to measure attitudes toward prisoners. Criminal Justice and Behavior.

[CR37] Merin O, Goldberg S, Steinberg A (2015). Treating terrorists and victims: A moral dilemma. The Lancet.

[CR38] Merin O, Goldberg S, Steinberg A (2015). Treating terrorists and victims: A moral dilemma. The Lancet.

[CR39] Ministry of Health (1996). *Patients’ Rights Law 1996*, pp. 2–3 https://www.health.gov.il/LegislationLibrary/Zchuyot_01.pdf [Hebrew].

[CR57] Ministry of Internal Security (1971). *Prison Order* [New Version]. https://he.wikisource.org/wiki/%D7%A4%D7%A7%D7%95%D7%93%D7%AA_%D7%91%D7%AA%D7%99_%D7%94%D7%A1%D7%95%D7%94%D7%A8 [Hebrew].

[CR40] Moorehead, C. (1998). *Dunant’s dream: War, Switzerland and the history of the Red Cross*. Harper Collins.

[CR41] OCHA (2010). *Humanitarian Principles*. http://ochanet.unocha.org/p/Documents/OOM_HumPrinciple_English.pdf.

[CR42] Ofran Y, Giryes SS (2004). To be a doctor in Jerusalem: Life under threat of terrorism. Annals of Internal Medicine.

[CR43] Ortet-Fabregat G, Perez J, Lewis R (1993). Measuring attitudes toward prisoners: A psychometric assessment. Criminal Justice and Behavior.

[CR44] Powell J, Harris F, Condon L, Kemple T (2010). Nursing care of prisoners: Staff views and experiences. Journal of Advanced Nursing.

[CR46] Prison Service (2024). *Prisoners’ report first quarter 2024*. https://www.gov.il/he/pages/report1_2024 [Hebrew].

[CR45] Prison Service (2019). *Commissioner’s Order no. 03.03.00 – Rules Regarding Security Prisoners* https://www.gov.il/he/departments/policies/nezivot_pkuda [Hebrew].

[CR47] Riba S, Reches H (2002). When terror is routine: How Israeli nurses cope with multi-casualty terror. Online Journal of Issues in Nursing.

[CR48] Rocks, S., & Schwarzwals, Y. (2000). *Social Psychology*, vol. 2, units 3,4. Tel Aviv: Open University [Hebrew].

[CR50] Shields KE, de Moya D, Gaines L (1997). Correctional health care nurses’ attitudes toward inmates. Journal of Correctional Health Care.

[CR51] Solell, P., & Smith, K. (2019). If we truly cared: Understanding barriers to person-centred nursing in correctional facilities. *International Practice Development Journal*, 9(2).

[CR52] State Comptroller (2015). *The Medical Care System for Prisoners in the Prison System – Annual Report 65C*, pp. 6–7 and 46. https://www.mevaker.gov.il/he/Reports/Report_290/6be0a5d6-9d84-4e6f-b923-ca97dc5c21fe/65C-205-ver-3.pdf.

[CR53] Tummala-Narra P (2005). Addressing political and racial terror in the therapeutic relationship. American Journal of Orthopsychiatry.

[CR54] Weiskopf CS (2005). Nurses’ experience of caring for inmate patients. Journal of Advanced Nursing.

[CR55] White AL, Larsson LS (2012). Exploring scope of practice issues for correctional facility nurses in Montana. Journal of Correctional Health Care.

[CR56] Yanko, A. (2023). The Health Minister gave the order and hospitals are moving terrorists to the Prison Service. *Ynet* (11 Oct. 2023). https://www.ynet.co.il/health/article/h1grfnv11a [Hebrew].

