# Supplementary material for: Attitudes of nurses, paramedics, and medics towards security prisoners: a cross-sectional study
Source: Health Justice. 2024 Apr 27;12:21. doi: 10.1186/s40352-024-00275-8 (PMC11055372; doi:10.1186/s40352-024-00275-8)
Supplement: Supplementary file 4 — Supplementary Material 4 [file 40352_2024_275_MOESM4_ESM.docx]

Attitudes toward prisoners scale

The following statements describe different attitudes toward prisoners in prisons and jails. There is no correct or incorrect answer, only the expression of an opinion. Please express *your* feelings toward each statement, indicating whether you (1) strongly disagree; (2) disagree; (3) undecided; (4) agree; or (5) strongly agree. Please indicate your opinion using the number that most accurately describes your personal opinion in the left-hand margin. Please respond to *all* the items.

Scale

| 1 | 2 | 3 | 4 | 5 |
| --- | --- | --- | --- | --- |
| Strongly disagree | Disagree | Undecided | Agree | Strongly agree |

1. Prisoners are different from most other people.
2. Few prisoners are really dangerous.
3. Prisoners never change.
4. Most prisoners are victims of circumstances and deserving of help.
5. Prisoners have feelings like anyone else.
6. Prisoners should not be trusted.
7. I think I would like many prisoners.
8. Difficult prison conditions make prisoners bitter.
9. Give a prisoner a finger and he will take the whole hand.
10. Most prisoners are unintelligent.
11. Prisoners require affection and praise like anyone else.
12. You should not develop high expectations of prisoners.
13. Prisoner rehabilitation efforts are a waste of time and money.
14. You never know when a prisoner is telling the truth.
15. Prisoners are no better or worse than other people.
16. You have to be constantly alert around prisoners.
17. In general, prisoners think and behave in similar ways.
18. If you show respect to a prisoner, he will respect you too.
19. Prisoners think only about themselves.
20. There are some prisoners I can trust with my eyes closed.
21. Prisoners are willing to accept logical explanations.
22. Most prisoners are too lazy to earn an honest living.
23. I would not mind living next door to a former prisoner.
24. Prisoners are heartless.
25. Prisoners are always trying to get something from someone.
26. Most prisoners’ values are the same as those of any of us.
27. I would never want one of my children to date a former prisoner.
28. Most prisoners are capable of love.
29. Prisoners just lack morals.
30. Prisoners should be held under strict and severe discipline.
31. In general, prisoners are fundamentally bad people.
32. Most prisoners can be rehabilitated.
33. Some prisoners are quite nice people.
34. I would like to be in the company of a few prisoners.
35. Prisoners only understand force.
36. If someone behaves well in prison, he should be released on parole.

Melvin, K.B., Gramling, L.K., & Gardner, W.M. (1985). A Scale to Measure Attitudes toward Prisoners. *Criminal Justice and Behavior*, 12(2), 241–253. https://doi.org/10.1177/0093854885012002006
