# Supplementary material for: Attitudes of nurses, paramedics, and medics towards security prisoners: a cross-sectional study
Source: Health Justice. 2024 Apr 27;12:21. doi: 10.1186/s40352-024-00275-8 (PMC11055372; doi:10.1186/s40352-024-00275-8)
Supplement: Supplementary file 1 — Supplementary Material 1 [file 40352_2024_275_MOESM1_ESM.docx]

16 April 2024

Dear Editor,

I am grateful for the opportunity to revise my article. I wish to thank the reviewers for the positive feedback and support. I appreciate the reviewer their educating and enlightening comments, which I found very useful to improving my manuscript.

**I have now finished applying all the reviewers' comments, as specified in the table below and marked in yellow in the text.**

**Reviewer 1**

| **Reviewer's comment** | **Our response** | **Page** |
| --- | --- | --- |
| Additional background information on security prisoners in Israel would be helpful for international readers. What are the demographics, security classifications, healthcare policies for this population? | Added information about the number of security prisoners.  Security classifications are not publicly available.  Added information about prisoners' medical care policy. | 3  -  4 |
| The authors note limitations of using a convenience sample recruited via social media, but more discussion of how this may impact generalizability would strengthen the paper. Future studies should aim for a more representative probability-based sample if possible. | Added explanation about the sample. | 17 |
| Public and healthcare worker perceptions may have shifted after major terror attack in October 7. The paper would benefit from discussion of how the sociopolitical climate regarding security prisoners may influence attitudes. | Added a section explaining the possibility of different attitudes following the 7 Oct. attack and resulting war, although the study was carried out before this. | 20 |
| While challenging given the brevity of a research report, integrating a few illustrative quotes from the qualitative portion of the study could enrich the discussion of the quantitative findings. | Added a few quotations from the qualitative study. | 18, 19 |

**Reviewer 2**

| **Reviewer's comment** | **Our response** | **Page** |
| --- | --- | --- |
| The abstract- a. The sample size should be reported under the Methods section, not the Results. | We have moved the mention of the sample size to the Methods section of the abstract. | 1 |
| b. The finding about the younger age group having lower average attitudes needs clarification. | Added an explanation of this finding. | 1 |
| The background- a. Updating the Israeli's prisoner population statistics to the latest available data | Added information about the number of security prisoners. | 3 |
| b. The background section could benefit from the addition of subtitles to help structure the literature review. Organizing the background information into clearly defined sections with relevant headings would improve the flow and readability of this part of the paper. | We have added subheadings in the Background section as requested. | 3-6 |
| 3. Method a. The study aim would be better placed in the Introduction or Background section, rather than the Methods section. Clearly stating the study objective upfront would help provide context for the reader before delving into the methodological details. | The aim has been moved to the end of the Introduction section. | 2 |
| b. Reorganizing the Methods section to clearly separate the details on participants, measures, and procedures would improve the flow and readability of this part of the paper. | The section about the participants has been moved into the Methods section from the Results section. The other parts of the Methods section already have subheadings describing the various measures used. | 7-10 |
| c. The measures: A referral to the supplementary material is missing (The questionnaire - Attitudes toward security prisoners) | The original questionnaire Attitudes Towards Prisoners (ATP) (Melvin et al., 1985) has been uploaded as supplementary material. | Supp. |
| 4. Results a. Presenting the table describing the sample characteristics (Table 1) in the Methods section (under participants) rather than the Results would help provide the reader with the relevant participant information upfront, before delving into the findings. | Table 1 has been moved to the Methods section, with the text discussing the participants. | 7-9 |
| b. The sample was predominantly Jewish (88.3%), with much smaller representations of Muslim and Christian participants. This imbalance in the sample composition could be problematic for the interpretation of the key finding that there was no significant difference in attitudes based on the staff's nationality. The paper could benefit from providing the complete percentages for the Muslim and Christian participants to better contextualize this result. | The percentages of Jewish and Arab (Muslim and Christian) participants are similar to the proportions in the general population of Israel. Added a comment explaining this. (73.2% Jewish, 21.1% Arab, 5.7% Other). | 17 |
| c. Include the complete percentages for all the demographic variables in Table 1, not just the partial percentages provided. For example, for the "Sex" variable, the percentage for females (56.2%) is given, but the percentage for males is missing. Additionally, ensure that the percentages for each variable add up to 100%. Some of the percentages currently presented do not total 100%, which could be confusing for the reader. | Table 1: Added data for the Male variable in the Sex category and for the Arab category in Nationality.  Where the percentages don't add up to 100%, it is due to one or more participants not answering the question. We have added a comment under Table 1. | 8-9 |
| 5. Discussion a. The claim that "No connection was found between the staff's nationality, whether Muslim, Jewish, or Christian, and their attitudes. This result is encouraging, because it indicates that the staff provides care in accordance with the Patients' Rights Law: without discrimination and without reference to the patient's characteristics" should be stated more cautiously. This conclusion appears to be based on the assumption that attitudes directly predict behavior, which may not always be the case. The authors may want to acknowledge this limitation and express the finding in a more tentative manner, as the relationship between attitudes and actual care provided is not definitively established by the current study. | The claim has been altered to: "… it indicates that the staff intends to provide care…".  Added a sentence in the Limitations section explaining that attitudes are not necessarily predictive of behavior, but are still worth examining. | 17  21 |
| b. The claim that "Staff put aside their personal characteristics, opinions, and emotions" towards security prisoners seems to be an assumption not directly supported by the data presented in the study. The research did not explicitly examine the emotional responses or level of emotional detachment of the healthcare staff when treating security prisoners. Therefore, the inference that staff were able to completely set aside their personal opinions and emotions should be stated more cautiously. | We have added quotations from the qualitative portion of the study, which do contain expressions of emotions. | 18-19 |
| c. The discussion contains some repetitive phrasing in lines 347-349. | This sentence has been revised | 18-19 |
| Limitations and future research a. The study focused on examining the attitudes of healthcare staff towards security prisoners, but did not assess their actual behaviors. This is an important limitation, as attitudes do not necessarily directly predict real-world behaviors. The paper could benefit from acknowledging this distinction and noting that further research is needed to understand the relationship between the staff's attitudes and the care they deliver to security prisoners. | Added a sentence in the Limitations section explaining that attitudes are not necessarily predictive of behavior, but are still worth examining. | 21 |
| b. The paper could benefit from acknowledging the significant imbalance in the representation of different nationalities in the sample. The sample was predominantly Jewish (88.3%), with much smaller proportions of Muslim and Christian participants. | The percentages of Jewish and Arab (Muslim and Christian) participants are similar to the proportions in the general population of Israel. Added a comment explaining this. (2023 figures: 73.2% Jewish, 21.1% Arab, 5.7% Other). | 17 |
| c. The literature suggests that a comprehensive assessment of attitudes should encompass three key components: cognitive (knowledge), affective (emotion), and behavioral (action) (Hornik, 1988). While the authors recommend follow-up studies to examine the knowledge component related to security prisoners, they do not mention the potential to also explore the emotional and behavioral aspects of the healthcare staff's attitudes in this context.  In addition, the emotional aspect may serve as an important mediating variable in the relationship between the staff's attitudes and their behaviors towards security prisoners, but this possibility is not explored in the current research. | We have added a statement regarding recommendation to study behavior regarding treatment of security prisoners. | 19 |
| d. As for further research, the study could benefit from exploring whether the participants' own experiences with terrorism or military service influence their attitudes towards security prisoners. | We have added a sentence with this recommendation. | 19-20 |
| e. As for further research, consider using a control group of healthcare providers who do not treat security prisoners for comparison. | We have added a recommendation for a direct comparison in future studies. | 19-20 |
